# Step-by-Step Metagenomics for Food Microbiome Analysis: A Detailed Review

**DOI:** 10.3390/foods13142216

**Published:** 2024-07-14

**Authors:** Jan Sadurski, Magdalena Polak-Berecka, Adam Staniszewski, Adam Waśko

**Affiliations:** Department of Biotechnology, Microbiology and Human Nutrition, Faculty of Food Science and Biotechnology, University of Life Sciences in Lublin, 20-704 Lublin, Poland; magdalena.polak-berecka@up.lublin.pl (M.P.-B.); adam.staniszewski@up.lublin.pl (A.S.); adam.wasko@up.lublin.pl (A.W.)

**Keywords:** bioinformatics, foodomics, metagenomics, microbiome, food

## Abstract

This review article offers a comprehensive overview of the current understanding of using metagenomic tools in food microbiome research. It covers the scientific foundation and practical application of genetic analysis techniques for microbial material from food, including bioinformatic analysis and data interpretation. The method discussed in the article for analyzing microorganisms in food without traditional culture methods is known as food metagenomics. This approach, along with other omics technologies such as nutrigenomics, proteomics, metabolomics, and transcriptomics, collectively forms the field of foodomics. Food metagenomics allows swift and thorough examination of bacteria and potential metabolic pathways by utilizing foodomic databases. Despite its established scientific basis and available bioinformatics resources, the research approach of food metagenomics outlined in the article is not yet widely implemented in industry. The authors believe that the integration of next-generation sequencing (NGS) with rapidly advancing digital technologies such as artificial intelligence (AI), the Internet of Things (IoT), and big data will facilitate the widespread adoption of this research strategy in microbial analysis for the food industry. This adoption is expected to enhance food safety and product quality in the near future.

## 1. Introduction

Consumer awareness has been on the rise in recent decades, leading to shifts in dietary preferences. This has resulted in an increased demand for more precise food research, as traditional microbiological culturing methods are limited in fully identifying microorganisms in food. These methods can only provide partial identification based on morphological and biochemical characteristics of culturable microorganisms, and they fall short in capturing unculturable microorganisms. Concerns have been raised regarding the incomplete microbial biodiversity picture presented by these techniques. Nevertheless, they continue to be the most commonly employed approaches in the food sector for gauging microbiological safety by identifying and describing the food’s microbiota.

In the 21st century, culture-independent methods have emerged, allowing the bypassing of microbiological culturing and its constraints [1]. These procedures, which are centered on nucleic acid assessment, involve PCR-DGGE (polymerase chain reaction–denaturing gradient gel electrophoresis), T-RFLP (terminal restriction fragment length polymorphism), and FISH (fluorescence in situ hybridization) [2]. The landscape of food research has been transformed by “omics” technologies, which produce extensive data on the collective attributes of a sample regarding microorganism structure, function, and growth dynamics. High-throughput screening (HTS) is integral to these technologies, enabling rapid and extensive measurements. Next-generation sequencing (NGS) typifies HTS technology.

NGS has notably decreased analysis costs, accelerated sequencing speed, and enhanced result quality. It is categorized into second- and third-generation sequencing methods. Technological advancements have steered a shift towards sequencing bacterial DNA as a mainstream approach in food microbiology research. NGS allows parallel mass sequencing of short sequence reads and individual long fragment sequencing. The emergence of HTS technology has spurred notable scientific development, culminating in the creation of four primary omics disciplines (genomics, transcriptomics, proteomics, and metabolomics) and their sub-disciplines (epigenomics, lipidomics, metallomics, etc.). By merging data from diverse omics areas, a new research discipline named “foodomics” has arisen, harnessing vast repositories of information [3].

“Foodomics” emerged as a distinct field in 2009, with a primary focus on investigating food and nutrition through omics technologies. The main goal of foodomics is to enhance food quality, thereby improving consumer health. The omics technologies employed in foodomics encompass nutrigenomics, proteomics, metabolomics, transcriptomics, and genomics [4]. Nutrigenomics specifically delves into how nutrients influence gene expression and aims to elucidate the interactions between bioactive food components and the genome at a molecular level, ultimately impacting gene expression. Nutrigenomics and nutrigenetics are often used interchangeably due to their close relationship [5]. Whereas nutrigenetics examines the correlations between single nucleotide polymorphisms (SNPs) and an individual’s response to dietary intake, nutrigenomics utilizes nutrigenetics to present a holistic perspective of an individual’s metabolism, with the intention of tailoring diets, preventing diseases, and mitigating life-threatening risks. This comprehensive understanding is facilitated by high-throughput sequencing techniques enabling a thorough examination of gene variations across the entire genome [6,7].

Proteomics delves into the study of proteins and their interactions within the cellular environment, which mirrors the constantly changing state of cells, tissues, and organisms. This field plays a crucial role in the identification of disease markers, as well as in the detection and accurate quantification of proteins, thereby enhancing our comprehension of disease causation. In contrast, metabolomics systematically identifies and measures all metabolites present in an organism.

Transcriptomics and genomics concentrate on the examination of nucleic acids found in biological specimens [8]. Transcriptomics specifically investigates gene expression at the RNA level, furnishing insights into the genetic makeup and functionality of genes across the entire genome to elucidate the molecular processes involved in specific biological functions. Genomics, on the other hand, is employed to conduct a thorough examination of an organism’s genetic material, aiding in the identification of species present in food, the determination of the abundance of such microorganisms, and the detection of contaminants such as foodborne pathogens [9]. The amalgamation of these omics technologies in the field of foodomics facilitates a comprehensive understanding of the composition, safety, and nutritional characteristics of food, thereby contributing to advancements in both food science and consumer health.

**Figure 1:**
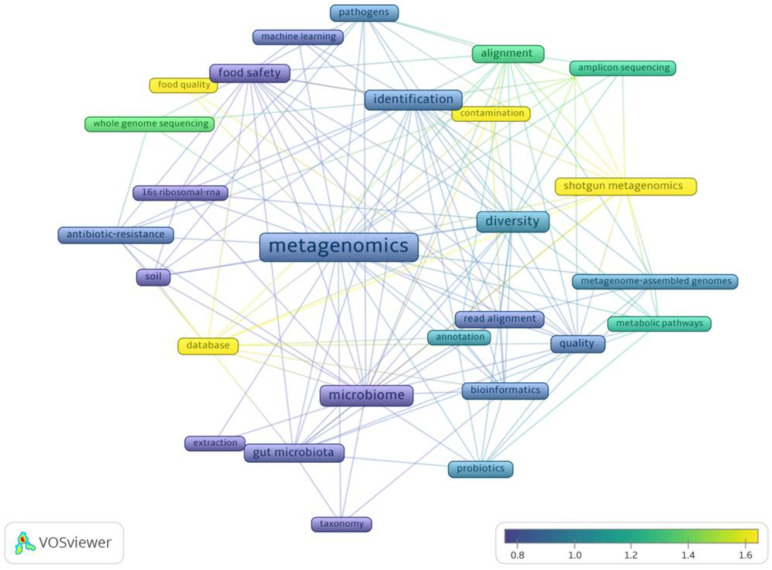
Co-occurrence of selected keywords in articles (2018–2023) using VOSviewer [10].

An analysis was conducted on publications related to food metagenomics from 2018–2023, using VosViewer (Figure 1) [10]. The bibliographic data of articles were retrieved from the Web of Science database. The analysis involved filtering results based on the keywords “food”, “metagenomics”, and “quality”. A co-occurrence network was established using 357 records from the past 5 years. The frequency of occurrences served as samples, resulting in 28 terms after excluding closely related ones. The input data for the network included full records and references cited. The size of each occurrence in the diagram corresponds to its frequency in publications. The color spectrum reflects the average normalized number of citations received by documents containing a specific term. Notably, articles featuring “shotgun metagenomics” and “database” garnered the highest number of citations, indicating researchers’ interest in research methodologies within the realm of “food quality”. Similarly, articles discussing untargeted sequencing details such as “amplicon sequencing”, “whole genome sequencing”, and “alignment” received significant attention, contrasting with the lower citation rate for articles mentioning “16S ribosomal RNA”, a targeted sequencing method. The limited occurrences and citations for terms such as “machine learning” and “extraction” suggest a research gap in the field of food metagenomics.

The objective of this review is to outline the individual processes involved in the analysis of food metagenomics, emphasizing its key aspects and presenting modern bioinformatic solutions.

## 2. Metagenomics

Metagenomics, a particular discipline within genomics frequently utilized in the field of food research, integrates genomic techniques with the principle of meta-analysis, which involves amalgamating and extrapolating findings from diverse studies through statistical approaches. The central emphasis of metagenomics lies in the sequencing and examination of the complete microbiota DNA, accompanied by metatranscriptomics that relies on cDNA (complementary DNA) sequencing [11]. Metagenomics obviates the need for the isolation and cultivation of microorganisms in vitro prior to DNA extraction. The focal point lies in the examination of the comprehensive genetic makeup of microorganisms existing in various natural specimens, such as food or the surrounding environment. Metagenomics encompasses scrutiny of the combined genome of microorganisms inhabiting a specified milieu, thus facilitating a comparative assessment of the microbiome through the utilization of NGS. The sequencing process can be directed towards a specific gene of interest, such as the 16S rRNA in bacteria, 18S in eukaryotes, or intergenic regions/internal transcribed spacers in fungi, or it can entail an untargeted approach known as “shotgun” sequencing, encompassing the sequencing of all genomes found within the sample, referred to as whole-metagenome sequencing (WMS) [12,13,14].

Sequencing can be executed through either of two methodologies: shotgun sequencing or targeted sequencing. Within the framework of shotgun sequencing, the DNA molecules undergo a random fragmentation process, resulting in small DNA pieces that are subsequently sequenced comprehensively. This particular approach is frequently applied in research endeavors pertaining to the characterization of microbial populations within metagenomic undertakings, aiming to discern microorganisms at a granular strain level. Conversely, targeted sequencing is exclusively concerned with the sequencing of a predetermined genomic region.

Shotgun sequencing, in comparison to targeted sequencing, provides additional insights into the functionality of the microbiome, its plasticity, and ongoing biological processes, as well as sequence variations and evolutionary variability. It also enables the identification of organisms at a higher taxonomic resolution [15], meaning at the lowest possible difference between organisms (taxonomic level). Access to complete genomes, rather than being confined to a solitary 16S/18S gene, is the reason for this phenomenon. The efficacy of sequencing the 16S gene persists in investigations of microbiota abundant in uncharacterized microorganisms. Research that focuses on sequencing for a specific microorganism or marker gene does not fall under the category of metagenomics, as it does not encompass the entirety of the genetic material in the specimen. Apart from the consistent reduction in sequencing expenses over time, one challenge of whole-metagenome sequencing (WMS) relates to the hardware prerequisites and intricate data interpretation. A strategy to address the high costs associated with WMS involves a two-stage approach. Initially, cost-effective targeted sequencing (16S rRNA) is carried out as a preliminary assessment, followed by untargeted sequencing on a chosen subset of specimens [13].

Metagenomic shotgun sequencing comprises both wet and dry phases. The wet phase denotes a laboratory procedure encompassing two steps: (i) acquiring and preserving samples and (ii) conducting sequencing. Conversely, the dry phase pertains to the computational handling of data derived from sequencing. Optimization of each phase in the analytical process is imperative, tailored to the specific material under investigation and the research goals.

## 3. Sampling and Storage

The most favorable approach for collecting samples entails striking a balance and the volume of samples needed, the frequency of repetitions, and the practical and financial viability of performing analyses [16]. The quantity of samples and repetitions significantly influences the precision, defined as the level of concordance between outcomes; accuracy, representing the level of adherence to the true condition; and the reproducibility of findings. The strategy for determining sampling sites with respect to the number of repetitions differs depending on the origin of the samples. Within the food sector, difficulties emerge concerning the optimal sample selection and the determination of repetition quantities. The microbiota of raw materials experiences rapid modifications throughout processing, even within brief time frames. Variations in the microbiota’s composition may be either straightforward to anticipate (e.g., milk after pasteurization) or unforeseeable, linked to other technological elements (e.g., frequency of equipment sanitation) [17]. The detailed procedure for sampling and DNA isolation from raw milk and cheese was developed by C. Barcenilla [18]. The uncertainty of temporal changes adds complexity to determining the optimal number of repetitions. Furthermore, the intricacy of the food production process presents additional hurdles. For instance, the production of cheese entails multiple phases carried out using distinct apparatus. Comprehending the production procedure is crucial for establishing the necessary quantity of sample collection points. Acquiring control samples in an environment with a variable microbiota composition, influenced by various external factors, can prove challenging. In such instances, it is advisable to substitute cross-sectional research, which compares the microbiota at a single time point, with prolonged investigations involving the analysis of samples from the same setting over an extensive duration. Prolonged studies do not depend on individual outcomes, which might deviate from the standard, and enable the removal of samples impacted by unfavorable alterations. If potential confounding variables cannot be ruled out, they should be factored into the comparative assessment [19]. A crucial aspect of collecting metagenomic samples is the preparation of detailed and standardized metadata. These are essential for comparative studies and result generation. Adopting a reporting standard enhances the quality, accessibility, and usefulness of information that can be stored in data repositories. The Genomic Standards Consortium (GSC) has proposed standards for the minimal information about genomic sequences (MIGS) and metagenomic sequences (MIMS). Additionally, these standards have been further detailed to include an environmental package comprising a set of measurements and observations describing the habitats from which the samples were collected. The environmental package includes sampling information such as geographical data on the location (country, region, latitude, and longitude), date of collection, environment, and material type [20]. It is advisable to gather a wide array of parameters, particularly the attributes specific to a given environment, in order to enhance the probability of establishing correlations between outcomes and a particular environmental factor [19]. A summary of sample fermented products along with the most commonly used DNA isolation kits and the required sample quantity is presented in Table 1.

## 4. Sequencing

Among the second-generation NGS technologies, the 454/Roche and Illumina/Solexa platforms are commonly utilized in metagenomic research. Key features of these technologies include the simultaneous production of millions of brief reads, decreased sequencing duration, reduced expenses in contrast to first-generation sequencing, and the capacity to acquire immediate outcomes. The advent of the third generation of NGS technologies, specifically long-read technologies such as PacBio and Oxford Nanopore, carries substantial implications for metagenomic investigations, especially in the process of genome assembly [36]. Long reads, abundant in valuable data, aid in de novo assembly and alignment with a reference genome (associating the sequenced genetic material with a recognized reference genome) [37]. These technologies facilitate the production of sequences measuring 10 kbp in length, achieving an accuracy rate of 85–87% for PacBio [38] and 88–94% for Nanopore [39]. This approach proves to be cost-efficient as it eliminates the need for extensive sample preparation procedures, thereby expediting the generation of outcomes in non-specialized laboratory settings. Nevertheless, a notable drawback of third-generation NGS lies in the inadequacy of bioinformatics resources tailored for the interpretation of lengthy sequences. Existing tools are predominantly optimized for the comparative assessment of precise data derived from short genetic sequences. Table 2 presents a summary of sample fermented products, target sequences, and the most commonly used sequencing technologies.

## 5. Bioinformatic Processing

**Figure 2:**
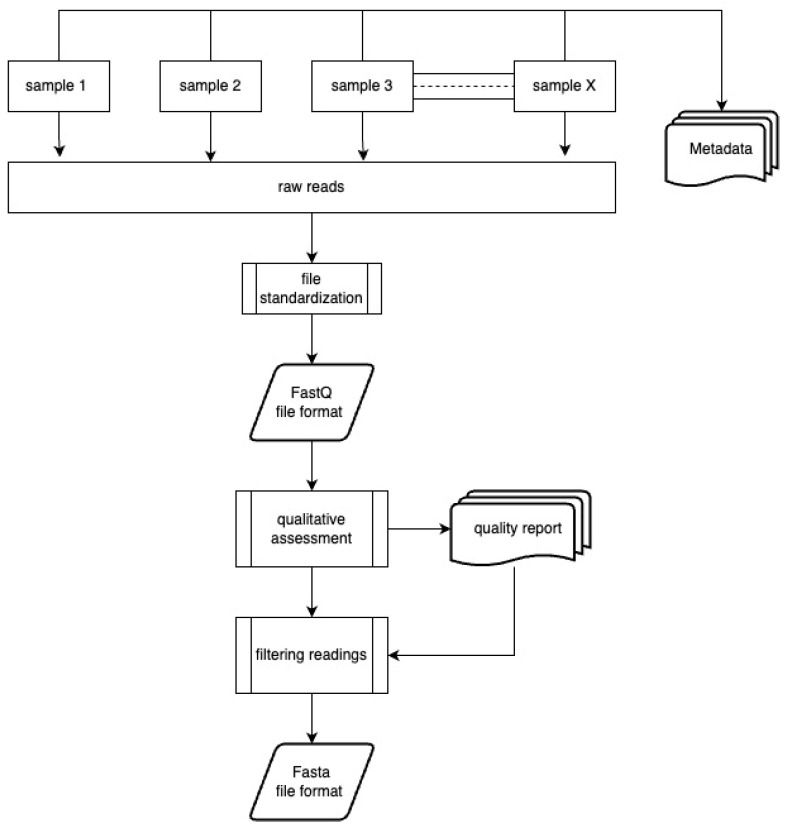
First-level analysis.

**Figure 3:**
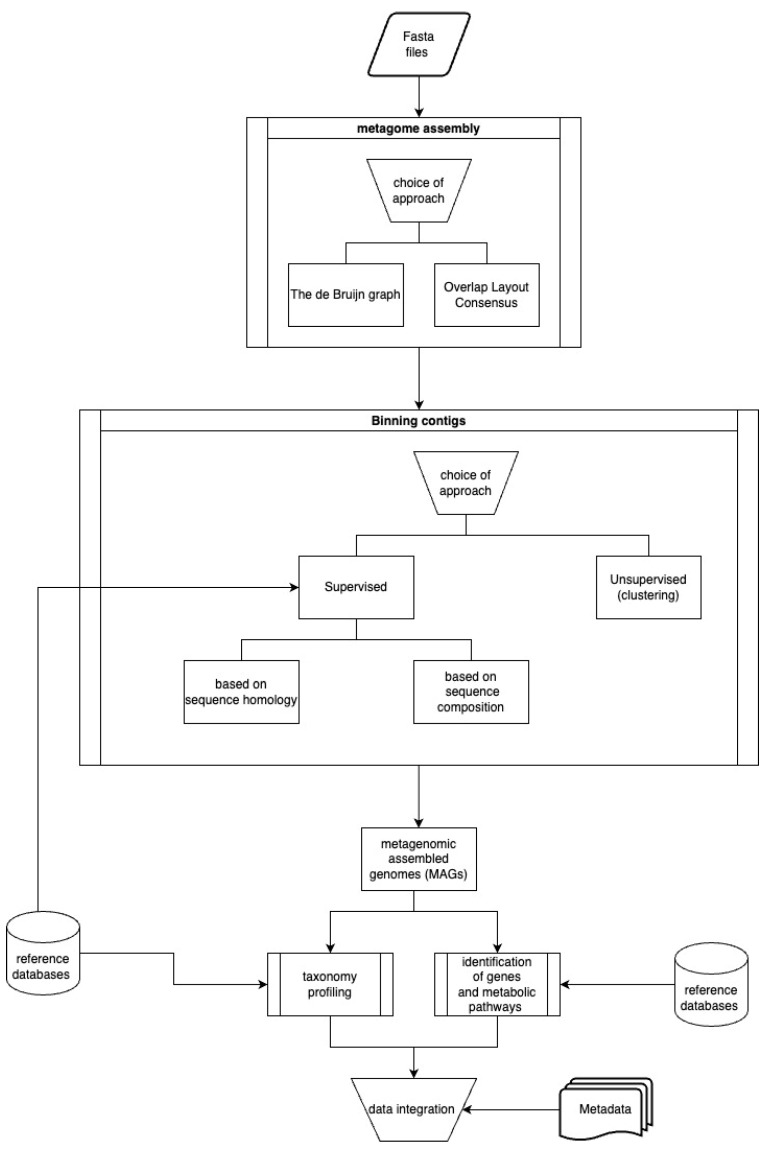
Second-level analysis and integrating results with metadata.

Working with sequences obtained using HTS involves a few manipulations of raw data performed by various programs in order to generate the desired final results. These procedures can be divided into 3 parts: (i) first-level analysis; (ii) second-level analysis, and (iii) integrating results with metadata. Figure 2 and Figure 3 present the subsequent stages of first- and second-level analyses.

### 5.1. Quality Assessment and Filtration of Readings

The objective of the initial processing stage is to enhance the overall quality of sequence files prior to their utilization in analytical procedures. Enhancing quality entails the filtration and elimination of low-quality sequences, along with the exclusion of any adapters that may be present. Quality evaluation is conducted at the individual base level utilizing the PHRED scale score, which indicates the likelihood of incorrect base assignment. Illumina-generated data typically exhibit high quality (Q30–40) at the onset of the read, gradually declining towards the read’s conclusion. Bases with a quality score below Q15–Q20 towards the end of the read are deemed insufficiently accurate for interpretation.

Depending on the employed sequencing methodologies, data are presented in various file formats. Assessment of the quality of reads can be conducted utilizing files in the FastQ formatting. Within the FastQ configuration, sequences and quality outcomes are delineated through individual ASCII characters. Each sequence comprises of four lines arranged one above the other. The initial line commences with the “@” symbol, followed by the sequence identifier (e.g., information on flow cell ID, read pairing). The succeeding line depicts the nucleotide sequence. The subsequent line initiates with the “+” symbol, succeeded by the identical sequence identifier as in the first line, denoting the conclusion of the sequence. The final line illustrates the quality of the sequence, with a solitary character encoding the quality (PHRED score) of a specific base within the sequence.

Files in the FASTQ format (Illumina, 454/Roche) can undergo qualitative evaluation through the utilization of the FASTQC software from https://www.bioinformatics.babraham.ac.uk/projects/fastqc/ (accessed on 13 July 2024). FASTQC is responsible for generating a total of ten visual representations aimed at assessing the quality of the file. The outcomes are stored in HTML format and are amenable to visualization through a web browser. Subsequent to this, the process of filtering results based on suitable quality parameters can be executed by employing trimming software such as SeqTrim [62], EA-Tools [63], and Trimmomatic [64].

Data acquired through the utilization of Nanopore technology are documented in the FAST5 file configuration, which encompasses raw signal information. The aforementioned data have the potential to be transformed into the FASTQ format by employing suitable software applications such as Guppy [39], Fast-Bonito [65], Causalcall [66], and NanoOK [67]. Guppy provides two distinct analysis models: rapid and high-precision. The mean quality of sequences produced by Nanopore falls within the range of Q7 to Q14, with the quality exhibiting fluctuations throughout the reading process. All numerical values in this context are highly significant.

PacBio sequences are archived in the Binary Alignment Map (BAM) format, a format that does not include annotations on sequence quality. The transformation to FASTQ format can be executed through software tools such as SAMTOOLS [68] or the SMRT portal [69]. Furthermore, the SMRT portal not only conducts demultiplexing (the inverse operation of multiplexing, which involves segregating signal components) but also eliminates hairpin adapter sequences from the reads and sieves out reads characterized by superior quality.

### 5.2. Contig Assembly De Novo

The process of de novo assembly involves the grouping of reads into contigs. Multiple techniques exist for determining the makeup of a multi-species microbial population from a set of sequence reads. When it comes to the methodology of metagenome assembly, the process is akin to piecing together individual whole genomes [68]. Different computational strategies are utilized for reconstructing the composition of microbial communities from a set of sequence reads, with the selection of approach being dependent on the objectives of the study. There are two primary methods for assembling contigs: (i) the utilization of a de Bruijn graph (DBG), and (ii) the alignment of overlapping OLC (overlap/layout/consensus) reads.

The prevalent approach utilized is based on the de Bruijn graph. This graph is formulated by disintegrating each read into overlapping fragments of a consistent length, known as k-mers. These k-mers establish the vertices and edges of the de Bruijn graph. Subsequently, the software traces a route within the graph, consequently ascertaining the accurate genomic sequence. The emergence of branches in the graph, which complicates the identification of the correct sequence, is attributed to sequencing inaccuracies, fluctuations in coverage, the existence of repetitive sequences, and various other structural variations [69].

**Figure 4:**
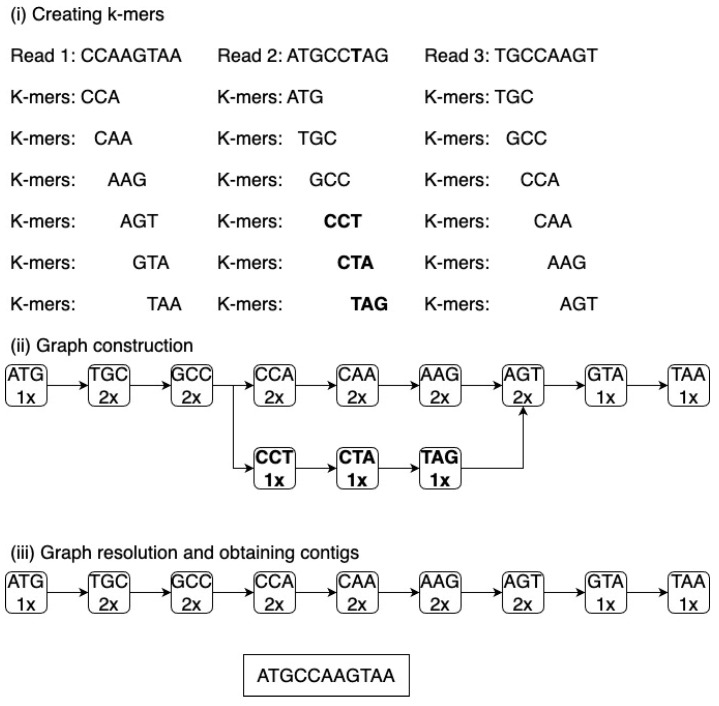
The de Bruijn graph. De Bruijn graph (**i**) reads are decomposed into k-mers by sliding a window of length k along the read. (**ii**) k-mers become nodes in the graph, and edges connect overlapping k-mers. Polymorphisms (highlighted in bold) create branches in the graph. The count of each k-mer’s occurrences is indicated (number above the k-mer). (**iii**) Contigs are built by traversing the graph between branching nodes. Algorithms may interpret branches differently; the example shown ignores low-coverage paths.

**Figure 5:**
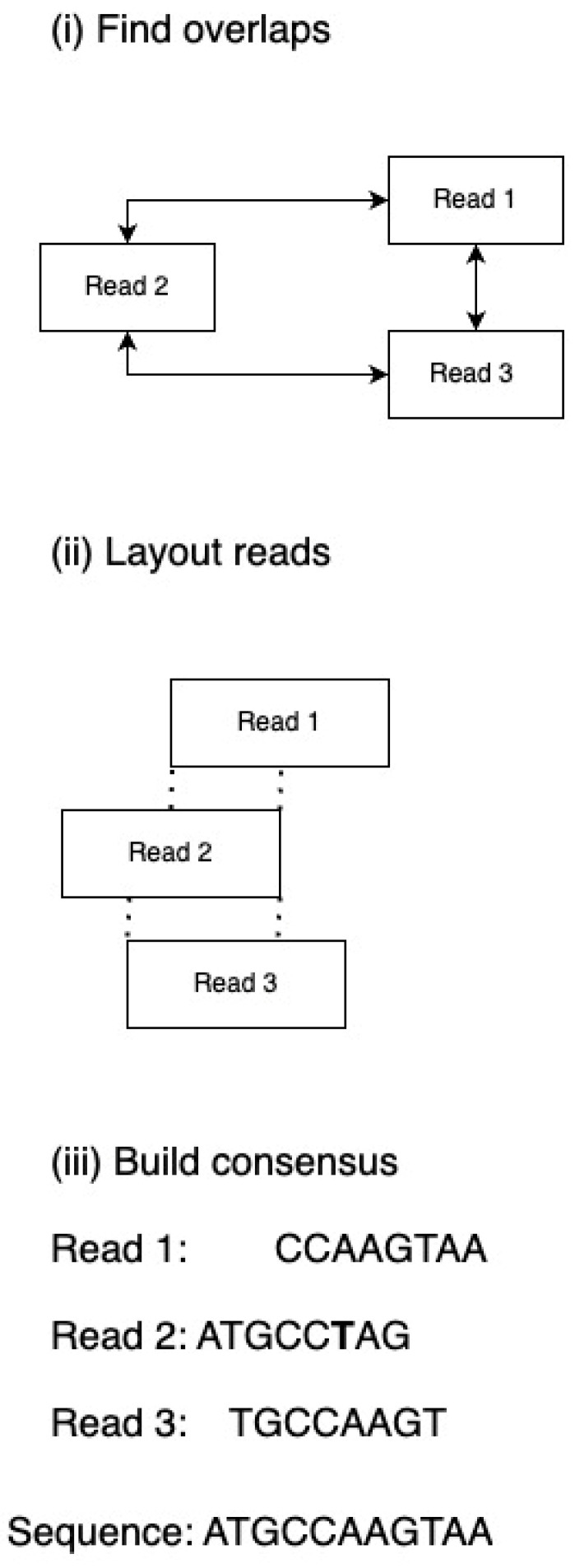
Overlap, layout, and consensus. Overlapping reads; (**i**) searching for overlapping read fragments. (**ii**) Layout reads determine the assembly of reads into contigs by considering coverage (dashed lines indicate overlapping fragments). (**iii**) The most probable nucleotides are selected for sequence construction.

A less frequently utilized approach entails the superimposition of reads, where the identification of overlapping sequences is achieved through the comparison of each read with all other reads. The overlapping reads are then categorized into contigs. Subsequently, a continuous sequence is established by choosing the most probable nucleotides from the overlapping contigs. However, a limitation of this method lies in the need to compare each read with every other read within the dataset, and in high-throughput sequencing (HTS) methodologies, the number of reads can reach millions. Figure 4 and Figure 5 illustrate the techniques utilized in contig assembly.

Assembling metagenomes poses a greater challenge compared to assembling individual genomes due to the absence of uniform sequence coverage throughout the genome. The coverage of each genome present in the metagenome is contingent upon the prevalence of microorganisms within the environment under study. Genomes that are poorly represented may experience fragmentation in cases where sequencing depth is insufficient [70]. The reduction in k-mer length utilized for graph construction may facilitate the reconstruction of less prevalent genomes, albeit with the drawback of heightened repeats that impede precise genome assembly [71]. An additional complexity emerges from the examination of closely related genomes exhibiting variations in individual genes or nucleotides, resulting in graph branching. The occurrence of branching within the graph contributes to the fragmented reconstruction of genomes.

Diverse strategies are being devised to tackle the challenges linked to metagenome assembly. Meta-IDBA [72] and RAMPART [73] employ varied k-mer lengths, eliminating the necessity of selecting a singular averaged k-mer length, thereby facilitating the assembly of genomes with diverse abundances. Additionally, RAMPART produces a concise overview for each assembly. In metagenome reconstruction, Meta-IDBA also accounts for irregular sequencing depth [74]. MetaSPAdes software allows hybrid metagenome assembly by utilizing short and long sequences acquired from different technologies [75]. Samples containing intricate microbiota compositions often encompass numerous closely related strains with varied abundances. Augmented sequencing depth enables their identification but demands substantial computational resources and time, which may prove inadequate. The MEGAHIT tool confronts this challenge by employing streamlined data structures to diminish memory requirements and expedite the analysis process when assembling intricate metagenomes [76]. A decentralized metagenome assembly framework harnesses Ray software [77] to allocate memory load across individual machines. Genovo diminishes analysis duration via advanced learning methodologies [78].

The initial categorization of reads into potential taxonomic clusters by comparing them to known genomes, and subsequently excluding them from the read collection, can optimize the process of constructing intricate metagenomes. MEGAHIT differentiates reads into well-defined and ambiguous categories based on their coverage relative to reference genomes. Even reads with limited coverage could be integrated into the metagenome assembly if they complement well-established contigs [76]. MetaCRAM [79] leverages Kraken [80], a k-mer-centric tool for taxonomic classification, to assign reads to reference genomes initially and eliminate any familiar sequences from the dataset before assembly. VICUNA software [81] facilitates the elimination of non-target reads through multiple sequence alignment (MSA) of the sequences.

Paired-end sequencing reads, encompassing both brief and extended fragments, offer significant advantages in the process of constructing individual genomes from scratch, yielding insights into the interconnections among divergent contigs and facilitating the formation of scaffolds. The utility of paired-end reads is less evident when dealing with metagenomic datasets. Several software applications designed for metagenome assembly adopt a methodology akin to that employed for individual genome assembly, involving the creation of scaffolds. Utilizing paired-end reads, tools such as MEGAHIT [78], BIGMAC [82], SPAdes [77], PRICE [83], and Omega [84] aim to identify and eliminate chimeric contigs resulting from the erroneous merging of distinct genomes, thereby enhancing the quality of the resultant assemblies. A comparative analysis of contig assembly software is presented in Table 3.

### 5.3. Contig Clustering

Complex metagenomes, characterized by extensive fragmentation resulting in thousands of contigs, pose challenges in determining the number of genomes present in the dataset and the assignment of contigs to specific genomes. The process of contig clustering aims to categorize these fragments into distinct groups representing individual species. This clustering endeavor leads to the reconstruction of components of intricate metagenomic genomes, referred to as metagenomic assembled genomes (MAGs). Subsequently, contigs belonging to each cluster are stored in separate files formatted in FASTA. FASTA-formatted data comprises sequences presented in a single line of text, with descriptions on subsequent lines initiated by the “>” symbol. Ideally, each file corresponds exclusively to a single genome.

Currently employed clustering methods can be divided into two categories: (i) supervised and (ii) unsupervised. Both methods assess the similarity between contigs and sets, subsequently transforming these similarities into assignments.

The method of supervised learning makes use of reference databases to categorize contigs into distinct taxonomic categories. Moreover, a distinction can be made within this method between sequence homology-based strategies and sequence structure-based strategies. Various tools that utilize sequence homology, such as PhymmBL [82] and BLAST [83], rely on vast and inclusive databases. Software programs such as PhyloPythiaS+ [84], EnSVMB [87], and Kraken [80] utilize k-mers to either compare sequences or generate patterns, ultimately reducing the time taken for analysis. The k-mer approach mandates the development of specific reference databases, while pattern generation necessitates the use of training files. The examination of metagenomes which encompass a multitude of genomes, lacking any reference databases, presents a significant challenge. The absence of reference genomes hinders the creation of training files. The substantial diversity of species within the files requires the generation of a larger quantity of patterns, consequently extending the time required for analysis.

Unsupervised clustering techniques aim to identify intrinsic variations within the examined dataset. The genomes of diverse organisms exhibit distinct arrangements of nitrogenous bases, which are manifested by discrepancies in the occurrence of k-mers [88]. The use of tetramers is considered to be most effective for clustering metagenomic information [89]. The process of grouping contigs is employed in various automated tools such as SCIMM [90] and MegaWatt [91], which rely on parameters related to species distribution and DNA sequence representation. Additionally, more advanced automated software such as MetaBAT2 [92], GroopM [93], and CONCOCT [94], as well as semi-automated algorithms that involve human evaluation, utilize contig clustering for analysis [95].

The hybrid method BMCCR (Binning Metagenomic Contigs using unsupervised Clustering and Reference databases) introduces a novel approach that integrates the benefits of two distinct methods, as outlined in a previous study [96].

### 5.4. Quality Assessments of MAGs

The quality of MAGs depends on the genome size of the species, its abundance in the environment, and the sequencing depth. Parameters determining MAG quality include completeness and the degree of genome contamination. CheckM [97] utilizes a set of marker genes, along with information about their positions in reference genomes, and then utilizes information about the co-localization of these genes in the studied genomes. The program initially places individual MAGs in the reference tree to adapt the set of marker genes to the specific lineage. 

**Figure 6:**
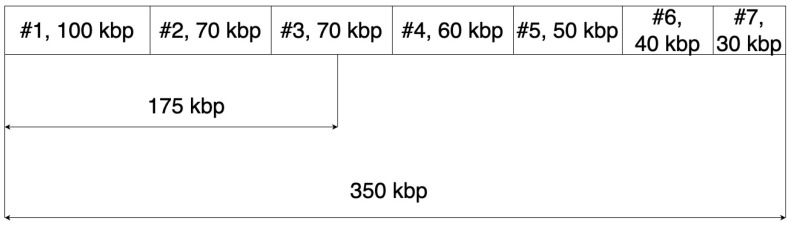
Construction of a MAG.

The optimal scenario entails acquiring a solitary contig within a document of comparable length to the taxonomically aligned genome. Nevertheless, the attainment of such a scenario is practically unattainable, hence, it becomes imperative to employ criteria that evaluate the assembly’s quality. One such criterion is N50, while another is L50. The quality of a MAG is deemed superior when N50 exhibits a higher value and L50 displays a lower value for the genomes being analyzed. The elucidation of parameters N50 and L50 is depicted in Figure 6.

Contigs sorted by length in descending order with a total length of 350 kbp. The N50 parameter for this sequence is the length of the contig located at the midpoint of the sequence–70 kbp. The L50 parameter is the position of the contig located at the midpoint of the sequence–3.

### 5.5. Defining and Analyzing the Pangenome

The pangenome denotes the entire array of genes discovered in diverse strains of a certain species, as ascertained through the utilization of comparative genomics examinations. This pangenome is structured hierarchically, comprising a genomic core, an accessory genome, and single genes. The genomic core consists of genes present in all strains of the species analyzed and is primarily responsible for crucial functions necessary for the microorganism’s survival [98], including those related to pathogenicity and virulence [99]. The accessory genome encompasses genes found in at least two strains but not in all [100], while single genes are those present in only one strain. Genes within the accessory genome or single genes can be acquired through processes such as horizontal gene transfer or mutations evolving in other genes. These genetic elements are crucial in facilitating adjustment to the surroundings, such as distinct biochemical pathways, harmfulness characteristics, and drug resistance [101]. The configuration of the pangenome can either be open or closed, depending on the likelihood of identifying novel gene families through the inclusion of genomes for comparative study. An open pangenome, when more genomes are added, tends to exhibit a growing diversity of gene families. Conversely, a closed pangenome remains stable in terms of the number of gene families present.

Pangenomic analysis entails a three-step procedure: (i) standardizing annotations, (ii) categorizing genes by orthology, and (iii) adjusting curves. Standardizing annotations in the initial stage aims to avoid misidentifying core genes as universal and incorrectly assigning universal genes to individual genes. This task typically utilizes genome annotation tools such as RAST [102] and Prokka [103]. At the second phase, a comprehensive table containing all orthologous genes is acquired. In this phase, software programs such as OrthoMCL [104] and Orthofinder [105] are utilized. This table enables the fitting of the specific curve that arises from permutations of all genomes at various positions during the third step. The alignment process incorporates Heaps’ law and the power law, while the alignment of the curve of the common genome and individual genes is accomplished through an exponential regression distribution. Table 4 showcases instances of pre-existing bioinformatics solutions that are capable of carrying out all three phases of analysis.

### 5.6. Taxonomic Profiling

The process of taxonomic profiling entails the assignment of operational taxonomic units (OTUs) to individual contigs. The primary objective of profiling is to ascertain the species composition of a metagenome and to estimate the representation of each species. There are two predominant strategies utilized for taxonomic identification: (i) aligning sequences using databases such as BLAST [83] and (ii) employing k-mers, exemplified by Kraken [80]. Kraken makes use of databases that house sequences fragmented into k-mers to seek out distinct fragments based on taxonomic categories, ranging from the lowest common ancestor (LCA) to the target species. The software dissects the queried contig into k-mers and subsequently assigns them to the most likely position in the reference taxonomic tree. A notable result of profiling is the prevalence of each OTU in the sample. The prevalence of individual species can be expressed in relative or absolute counts of OTUs [112].

### 5.7. Construction of Phylogenetic Trees from Metagenomic Data

The procedure for constructing a phylogenetic tree is a multi-step process. It consists of the following: (i) orthology prediction (orthologous genes, i.e., genes whose relationships will reliably reflect species relationships), (ii) alignment, (iii) identification of outliers, (iv) site filtering, and (v) phylogenetic inference.

There are bioinformatics solutions that perform all these steps within their scope. These include: PhyloPhlAn [113], PhyloSift [114], ezTree [115], GToTree [116], and AMPHORA [117], which rely on the analysis of specific genes, their sets, or specific regions in the genome [118]. Additionally, there are algorithms responsible for specific stages of the analysis, such as algorithms for multiple-sequence alignment (MSA): MUSCLE [119], MAFFT [120], T-Coffee [121], OPAL [122], PASTA [123], and UPP [124], and phylogenetic reconstruction algorithms such as FastTree [125,126], RAxML [125,127,128], ASTRAL [129], ASTRID [130], and IQ-TREE [131]. Each algorithm performs analyses separately or sequentially, requiring the researcher to have substantial knowledge in identifying appropriate targets, parameters, and steps of computational phylogenetics. A detailed review on obtaining phylogenetic trees using a non-automated multi-step method has been presented by P. Kapli in his article [132].

The separate and human-monitored execution of these processes is not feasible, especially when a large quantity of genomes are collected and analyzed together. Efficient algorithms have been suggested, such as those utilizing a small number of representative marker genes, such as the multilocus sequence typing (MLST) method or core genes at the species level. Computational MLST can function rapidly by employing only five to ten loci for each species. An example of an MLST-based program is chewBBACA. chewBBACA is capable of constructing phylogenetic trees using whole-genome MLST (wgMLST) or core genome MLST (cgMLST) [133]. Nevertheless, this is achieved at the cost of significantly reduced accuracy in phylogenetic placement. Pangenome-based profiling, exemplified by Roary [111], excels in accurate phylogenetic modeling at the species level but cannot be applied broadly to higher clades. Phylogenies that isolate strains and incorporate thousands of reference genomes from various species—or at least those most closely related to new sequences—result in a more precise depiction of microbial population structures and traits, aiding in more precise taxonomy. 

### 5.8. Determining Gene Functions and MAG Metabolic Profiles

For fragmented yet of high quality MAGs, it is possible to establish a metabolic profile. The process of genome annotation can be conducted using two primary methodologies. One strategy entails the identification of genes along with their respective functions; however, this approach is constrained by a vast reservoir of genes in databases that lack characterization. The alternative approach involves a translational search for proteins affiliated with specific functional categories. Databases such as UniProt [134] and KEGG [135] offer both annotation services and insights into the categorization of proteins into distinct functional clusters and metabolic pathways. The annotation outcomes are typically displayed graphically or in the format of a TSV file containing numerical data regarding the presence or absence of particular pathways. A notable limitation in metabolic profiling lies in the absence of scrutiny of accessory genes, thereby leading to the recognition and quantification of primary metabolic pathways within a comparable framework. Despite variations in microbiological and environmental compositions, samples demonstrate analogous functional attributes [136].

Moreover, the process of identifying genes can be further advanced through the utilization of specific software designed for identifying virulence genes, such as MetaPhinder [105], or genes associated with antibiotic resistance [106].

### 5.9. Integrating Metagenomic Data with Metadata

The obtained data on the microbiological characteristics of the metagenome are processed using statistical tools for interpretation and exploration of correlations with the collected metadata of the samples. R, a popular programming language, is commonly employed for statistical analysis. It encompasses a range of packages dedicated to both metagenomics and genomics, which can be adapted for metagenomic purposes. Detailed packages have been described by Calle in his review article [112].

## 6. Software for Comprehensive Metagenomic Analyses

There exist pre-configured software bundles with predetermined parameters for every phase of analysis. These are characterized by a higher level of user-friendliness attributed to a graphical user interface (GUI), thereby obviating the necessity of employing a text-based interface. In addition, these bundles encompass an exhaustive array of tools essential for multifaceted data analysis, visualization, and interpretation. The setup process of such bundles is streamlined through an integrated automated installer that is compatible with various platforms. Illustrative instances of such software bundles encompass Parallel-Meta Suite [82] and EPA-ng [137]. One drawback of these solutions pertains to the limited adaptability in adjusting parameters at specific analysis stages, a factor that could prove pivotal in achieving the desired outcomes.

**Figure 7:**
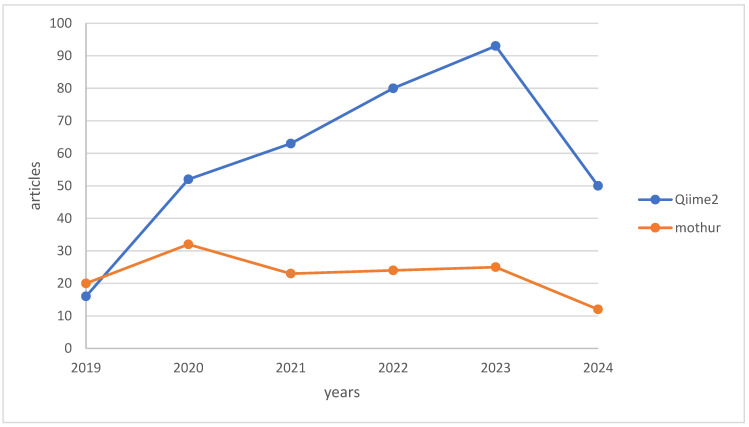
Comparison of citation counts: QIIME2 vs. Mothur.

Mothur [138] and Qiime2 [139] are widely used solutions in bioinformatics analysis, offering flexibility in parameter adjustments. Mothur, initiated in 2009 by Dr. Patrick Schloss at the University of Michigan, provides an integrated platform for ecological research through a command line interface, while Qiime2, designed with a plugin system, allows operation via API, graphical interface, and command line for decentralized use. Figure 7 illustrates a graph presenting the citation frequencies of Qiime2 and Mothur in scholarly articles from the last 5 years, based on data obtained from PubMed on 7 July 2024.

## 7. Conclusions

“Foodomics” represents a novel research approach within the realm of food studies, demonstrating considerable potential for application in the realm of food production. This methodology facilitates a profound examination of food microbiota through the utilization of contemporary, cost-effective, and efficient DNA sequencing techniques. Through metagenomic scrutiny, a precise taxonomic classification can be achieved, pinpointing individual species and strains. Furthermore, this approach allows the identification of specific gene functions that could influence the production process and, consequently, the sensory attributes of the final product. The acquisition of dependable and replicable outcomes necessitates the consideration of various factors during the design phase of studies. Fundamental elements encompass the approach to sample collection, storage, and pre-sequencing preparation, alongside subsequent bioinformatic scrutiny. The formulation of optimal and consistent sampling techniques, coupled with the meticulous documentation of pertinent environmental variables specific to distinct production procedures, is imperative. The realm of bioinformatic solutions geared towards comprehensive microbiota analysis is in a perpetual state of evolution, being continually generated, refined, and enhanced. At present, a majority of existing programs lack a user-friendly graphical interface, thereby heightening initial operational complexities. In the forthcoming years, bioinformatic solutions featuring intuitive graphical interfaces will emerge. Software integrating deep learning methodologies will streamline the analysis timeframe and diminish hardware prerequisites. Nevertheless, systems leveraging deep learning necessitate a training phase, mandating substantial resources and time investments. The efficacy of bioinformatic tools is contingent upon the breadth of reference databases, underscoring the need for their continual expansion to encompass newly unearthed genes and proteins. The evolution of foodomics towards heightened accessibility and efficacy paves the way for its integration into the commercial sphere. The employment of foodomic technologies in production monitoring will aid in refining the production pipeline by pinpointing and eradicating avenues of entry for pathogenic microorganisms, while simultaneously overseeing the growth of beneficial microorganisms. In contrast to traditional microbiological methodologies, outcomes will be expedited (independent of microbial incubation periods) and will furnish a more precise depiction of the scrutinized metagenome composition.

## 8. Glossary

- ASCII (American Standard Code for Information Interchange) is a character encoding system utilized in computers and communication devices to symbolize textual characters, with each character being allocated a distinct numerical value represented as an integer within the range of 0–127.
- FASTA is a file format employed for the storage of DNA, RNA, and protein sequences.
- Deep learning is a division of artificial intelligence (AI) that concentrates on the development and training of neural networks capable of learning and executing tasks automatically, without the need for explicit programming.
- HTML (Hypertext Markup Language) is a markup language utilized for the construction of websites, serving as the foundational language for structuring and presenting content on the web.
- HTS (high-throughput screening) involves high-throughput techniques for screening vast quantities of substances, leveraging automation and miniaturization to analyze numerous substances simultaneously. Various detection methods are employed, such as chemical reactions, absorbance, fluorescence, and bioluminescence, to identify the substances being tested.
- Kbp (kilobase pair) is a unit of measurement in molecular biology equivalent to 1000 nucleobase pairs.
- A k-mer is a nucleotide sequence of length k in DNA or RNA, comprising any of the four nucleotides: adenine, guanine, cytosine, and thymine in DNA or uracil in RNA.
- Contig refers to a continuous series of nucleotides within the genome, generated by amalgamating DNA sequence reads.
- MAG (metagenome-assembled genome) denotes a genome reconstructed from the combined genetic material present in a sample containing taxonomically diverse organisms from a specific environment.
- NGS (next-generation sequencing) encompasses advanced sequencing methodologies that facilitate rapid and simultaneous reading of multiple DNA fragments.
- OTU (operational taxonomic unit) is a taxonomic grouping for nucleotide sequences based on their sequence similarity.
- PHRED is a computational tool that evaluates the quality of DNA sequences acquired during sequencing, providing a probability estimation of errors in reading specific nucleotides. The resultant quality assessment, known as the PHRED score, is expressed as a numerical value on a logarithmic scale (0, 20, 40, 60), where higher values indicate greater accuracy in reading.
- Scaffolds denote extended sequences comprising ordered and linked contigs, representing a segment of the genome not assigned to a particular chromosome.
- TSV (tab-separated values) is a text file format where values are delimited by the tab character (TAB), facilitating the storage and transmission of data in tabular form.
- WMS (whole-metagenome sequencing) encompasses complete sequencing of the metagenome, enabling the analysis of all genetic material within a metagenomic sample.

## Figures and Tables

**Table 1:** Fermented products and methods of DNA isolation.

Product	Sampling Detail	DNA Isolation	References
Cheese	1 g sample	PowerFood Microbial DNA Isolation Kit	[21,22,23]
Kombucha	50 mL sample	PureLink Microbiome DNA Purification Kit	[24,25,26]
Kefir	3–5 g sample	DNeasy PowerSoil Kit	[27,28]
Yogurt	10–20 mL sample	QIAamp Fast DNA Stool Mini Kit	[29,30]
Kimchi	3 mL sample	QIAamp Fast DNA Stool Mini Kit	[31,32,33]
Sauerkraut	1.2 mL sample	FastDNA™ S PIN kit for Soil	[34,35]

**Table 2:** Fermented products and methods of sequencing.

Product	Target	Sequencing Platform	References
Cheese	16S rRNA V3-V4 16S rRNAV4 MGS	Illumina MiSeq Illumina MiSeq Illumina HiSeq	[40,41,42,43]
Kombucha	16S rRNA V1-V9 MGS MGS 16S rRNA V1-V9	Oxford Nanopore Technologies MinION Illumina HiSeq Illumina Novaseq 6000 Illumina NextSeq 500	[44,45,46,47]
Kefir	MGS 16S rRNA V3-V4	Illumina HiSeq Illumina MiSeq	[48,49,50,51]
Yogurt	MGS 16S rRNA V2, V4, V6, V7, V8, V9 16S rRNA V2-4-8, V3-7-9	Illumina HiSeq Ion GeneStudio S5 Ion Torrent PGM	[52,53,54,55]
Kimchi	16S rRNA V3-V4 16S rRNA V1-V3	Illumina MiSeq Roche 454 GS-FLX Plus	[56,57]
Sauerkraut	16S rRNA V3-V4	Illumina MiSeq Illumina NovaSeq	[58,59,60,61]

**Table 3:** Programs assembling contigs.

Program	Method (dBG—de Bruijn Graph; OLC—Overlap Layout Consensus)	Characteristic Feature	Publications
Genovo	OLC	It employs deep learning; randomly selects contigs for matching reads	[78]
IDBA-UD	dBG	It breaks down the graph locally at each depth.	[72,74]
MEGAHIT	dBG	K-mers split based on identification with reference genomes.	[76]
Omega	OLC	Scaffolding using long reads; unmatched contigs are grouped based on coverage.	[85]
Price	Hybrid	Identical reads are assembled first, followed by less similar ones.	[86]
Ray	dBG	Distributed program connected to the network; profiles the microbiome based on unique labeled k-mers.	[77]
SPAdes	dBG	The metaSPAdes extension utilizes stream processing to resolve the graph.	[75]

**Table 4:** Programs designed for the analysis of pangenomes.

Software	Orthology Analysis	Pangenome Construction	References
BPGA	CD-HIT, OrthoMCL	Power-law regression	[106]
EDGAR 2.0	Score ratio values	Heaps’ law	[107]
GET_HOMOLOGUES	COGtriangles, OrgoMCL	Plot_pancore_matrix.pl	[108]
PanWeb	PGAP	PGAP	[109]
PGAP	MultiParanoid, Gene Family	Heaps’ law	[110]
Roary	CD-HIT, BLAST, MCL	(Not mentioned)	[111]

## Data Availability

No new data were created or analyzed in this study. Data sharing is not applicable to this article.
